# Na^+^/H^+^ Exchanger 3 Is Expressed in Two Distinct Types of Ionocyte, and Probably Augments Ammonia Excretion in One of Them, in the Gills of the Climbing Perch Exposed to Seawater

**DOI:** 10.3389/fphys.2017.00880

**Published:** 2017-11-02

**Authors:** Xiu L. Chen, Biyan Zhang, You R. Chng, Jasmine L. Y. Ong, Shit F. Chew, Wai P. Wong, Siew H. Lam, Yuen K. Ip

**Affiliations:** ^1^Department of Biological Sciences, National University of Singapore, Singapore, Singapore; ^2^Natural Sciences and Science Education, National Institute of Education, Nanyang Technological University, Singapore, Singapore; ^3^NUS Environmental Research Institute, National University of Singapore, Singapore, Singapore

**Keywords:** air-breathing fish, euryhaline, iono-osmoregulation, acid-base balance, ammonia

## Abstract

The freshwater climbing perch, *Anabas testudineus*, is an euryhaline teleost and an obligate air-breather with the ability to actively excrete ammonia. Members of the Na^+^/H^+^ exchanger (NHE) family help maintain intracellular pH homeostasis and ionic balance through the electroneutral exchange of Na^+^ and H^+^. This study aimed to obtain, from the gills of *A. testudineus*, the full cDNA coding sequence of *nhe3*, and to determine the effects of exposure to seawater or 100 mmol l^−1^ of NH_4_Cl in fresh water on its mRNA and protein expression levels. Efforts were also made to elucidate the type of ionocyte that Nhe3 was associated with in the branchial epithelium of *A. testudineus*. The transcript level and protein abundance of *nhe3*/Nhe3 were very low in the gills of freshwater *A. testudineus*, but they increased significantly in the gills of fish acclimated to seawater. In the gills of fish exposed to seawater, Nhe3 was expressed in two distinct types of seawater-inducible Na^+^/K^+^-ATPase (Nka)-immunoreactive ionocytes. In Nkaα1b-immunoreactive ionocytes, Nhe3 had an apical localization. As these ionocytes also expressed apical Rhcg1 and basolateral Rhcg2, which are known to transport ammonia, they probably participated in proton-facilitated ammonia excretion in *A. testudineus* during seawater acclimation. In Nkaα1c-immunoreactive ionocytes, Nhe3 was atypically expressed in the basolateral membrane, and its physiological function is uncertain. For *A. testudineus* exposed to NH_4_Cl in fresh water, the transcript and protein expression levels of *nhe3*/Nhe3 remained low. In conclusion, the branchial Nhe3 of *A. testudineus* plays a greater physiological role in passive ammonia transport and acid-base balance during seawater acclimation than in active ammonia excretion during environmental ammonia exposure.

## Introduction

Na^+^/H^+^ exchangers (NHEs/Nhes) belong to the solute-carrier 9 family and are integral membrane proteins that regulate pH homeostasis, cell volume, and ionic balance by transporting Na^+^ in exchange for H^+^ in a 1:1 stoichiometry (Fliegel and Dibrov, 1996; Counillon and Pouyssegur, 2000). In mammals, 9 isoforms of NHE (NHE1–9) have been identified with each isoform exhibiting considerable heterogeneity in their tissue expression and membrane localization (see Orlowski and Grinstein, 2004; Donowitz et al., 2013 for reviews). Around 70% of Na^+^ and 80% of ${\text{HCO}}_{3}^{-}$ in mammalian nephrons are reabsorbed from the lumen by active transport in proximal tubules (Wagner et al., 2004), and NHE is the main apical Na^+^ transporter present in the proximal tubular cells (Capasso et al., 2005). NHE2, NHE3, and NHE8 are expressed in the brush-border membrane of the proximal tubules, and approximately half of the entire apical NHE activity is facilitated by NHE3 (Choi et al., 2000).

Compared to terrestrial vertebrates, euryhaline teleosts have evolved sophisticated ionoregulatory and osmoregulatory mechanisms to counter the effects of salinity and osmotic stress (see Hwang et al., 2011 for a review). In fishes, the gill is the major osmoregulatory organ responsible for ionoregulation and acid-base balance (Evans et al., 2005), accounting for more than 90% of the transfer of acid-base equivalents (H^+^ and ${\text{HCO}}_{3}^{-}$) (Perry and Gilmour, 2006). Members of the Nhe family are key transporters in the ionocytes of fish gills (see Hwang and Lee, 2007; Hwang et al., 2011; Dymowska et al., 2012; Kumai and Perry, 2012 for reviews), as they play a critical role in transmembrane transport of ions driven by a combination of the inwardly-directed Na^+^ gradient and the outwardly-directed H^+^ gradient (Paillard, 1997). Activities of several Nhe isoforms are known to be triggered by a drop in intracellular pH which results in an increased rate of H^+^ excretion (Claiborne et al., 2002). Recent studies have shown that branchial Nhe isoforms can participate in Na^+^ absorption in freshwater teleosts and H^+^ secretion in some marine species (see Hwang et al., 2011 for a review). Furthermore, Nhe3 can be co-expressed with Rhesus family C glycoprotein 1 (Rhcg1), to form a metabolon that secretes H^+^ to “trap” NH_3_ excreted across the apical membrane of ionocytes (Wright and Wood, 2009; Hwang et al., 2011; Ito et al., 2013).

The climbing perch, *Anabas testudineus*, is classified under Order Perciformes and Family Anabantidae. It is a euryhaline freshwater teleost found in tropical Asia. It is highly tolerant of adverse water conditions (Pethiyagoda, 1991) and can survive in a broad range of salinities (Chang et al., 2007). It possesses a pair of accessory breathing organs (ABO) in the upper compartment of the branchial chambers for air-breathing (Graham, 1997). Being an obligate air-breather, *A. testudineus* normally rises to the water surface at regular intervals to gulp air, which is channeled to the ABO for gaseous exchange. In addition, *A. testudineus* exhibits the remarkable ability to actively excrete ammonia when exposed to high concentrations of NH_4_Cl, and can survive in fresh water containing 100 mmol l^−1^ of NH_4_Cl for at least 6 days (Loong et al., 2011; Ip et al., 2012a,b). By contrast, many teleosts would perish after a few hours of exposure to 1–5 mmol l^−1^ of NH_4_Cl.

After 6 days of exposure to seawater or 100 mmol l^−1^ NH_4_Cl in fresh water, the mRNA expression levels of *Na*^+^*:K*^+^*:2Cl*^−^ *cotransporter1a* (*nkcc1a*), *cystic fibrosis transmembrane conductance regulator* (*cftr*) and the protein abundance of Nkcc1 increase significantly in the gills of *A. testudineus* (Loong et al., 2011; Ip et al., 2012b). For *A. testudineus* exposed to seawater, Cl^−^ enters the branchial ionocytes through basolateral Nkcc1 and is subsequently excreted through apical Cftr, while Na^+^ that has entered the ionocytes exits to the external environment through the paracellular route (Loong et al., 2011; Ip et al., 2012b), which are in agreement with the classic model for branchial NaCl excretion in marine teleosts (Evans et al., 2005; Hwang and Lee, 2007; Hwang et al., 2011). For *A. testudineus* exposed to NH_4_Cl, it has been proposed that ${\text{NH}}_{4}^{+}$ is transported, in substitution of K^+^, from the plasma into ionocytes through the basolateral Nkcc1 (Loong et al., 2011), and exit the apical membrane through the Rh-associated glycoprotein (Rhag) down a favorable electrochemical potential generated by the excretion of Cl^−^ and/or ${\text{HCO}}_{3}^{-}$ through the apical Cftr (Ip et al., 2012b; Chen et al., 2017). Three branchial Nka α-subunit isoforms (Nkaα1a, Nkaα1b, and Nkaα1c) are known to be expressed in the gills of *A. testudineus* under various conditions (Ip et al., 2012a). Nkaα1a is the freshwater-isoform important for ion absorption, while both Nkaα1b and Nkaα1c are seawater-isoforms essential for ion secretion (Ip et al., 2012a; Ching et al., 2013). Nkaα1c is regarded as the ammonia-isoform possibly involved in active ${\text{NH}}_{4}^{+}$ excretion in the gills of ammonia-exposed *A. testudineus* (Ip et al., 2012a; Chen et al., 2017). It has been established that the active extrusion of Na^+^ and Cl^−^ in the gills of *A. testudineus* during seawater acclimation and the active excretion of ${\text{NH}}_{4}^{+}$ during environmental ammonia exposure may involve similar kinds (i.e., Nka, Nkcc1, and Cftr) of transporter but different types (i.e., seawater-inducible or ammonia-inducible) of Nkaα-immunoreactive ionocyte (Ip et al., 2012a).

In fishes, ionic regulation and acid-base balance are intricately linked because of the tight coupling of apical Na^+^/H^+^ exchange through Nhe3 in branchial ionocytes (Evans et al., 2005; Perry and Gilmour, 2006). Ionocytes in fish gills respond to changes in pH and salinity of the external environments through the concurrent activation of acid-base regulatory and osmoregulatory mechanisms, and Nhe3 is implicated as the relevant transporter (Furukawa et al., 2011; Liu et al., 2016). Furthermore, acid-base regulation, ammonia excretion, and osmoregulation have been shown to be closely linked in the seawater-inducible ionocytes of medaka acclimated to seawater (Liu et al., 2013, 2016). Notably, *A. testudineus* is known to increase ammonia production and excretion during acclimation from fresh water to seawater (Chang et al., 2007), but there is currently a dearth of information on the possible roles of Nhe3 in Na^+^ absorption in fresh water, and in acid-base balance as well as ammonia excretion in seawater. Furthermore, it is unclear whether Nhe3 takes part in active ammonia excretion in *A. testudineus* during exposure to high concentrations of NH_4_Cl in fresh water. Therefore, this study was undertaken to obtain the complete cDNA coding sequence of *nhe3* from the gills of *A. testudineus*, and to determine the effects of exposure to seawater or environmental ammonia in fresh water on the branchial mRNA expression level of *nhe3*. Based on the deduced Nhe3 sequence, a custom-made anti-Nhe3 antibody was made to determine the protein abundance of Nhe3, and to examine its cellular and subcellular localization, in the gills of *A. testudineus*. As ammonia production and excretion increase in *A. testudineus* acclimated to seawater (Chang et al., 2007), which may interfere with acid-base balance, the hypothesis tested was that exposure to seawater would up-regulate the mRNA and protein expression levels of *nhe3*/Nhe3 in the gills. Furthermore, it was hypothesized that Nhe3 would have an apical localization in certain seawater-inducible Nkaα-immunoreactive ionocytes, whereby it could mediate Na^+^/H^+^ exchange with the external medium. It has been established that *A. testudineus* alkalinizes the external medium when exposed to ammonia in fresh water (Ip et al., 2012b). Hence, we hypothesized that ammonia exposure would have minimal effects on the gene and protein expression levels of *nhe3*/Nhe3 in its gills.

## Note on abbreviations

Ammonia refers to both NH_4_^+^ and NH_3_, while NH_4_^+^ and NH_3_ denote ammonium ion and unionized molecular ammonia, respectively. Two different kinds of abbreviations were used in this study as the accepted abbreviations of genes and proteins of teleosts (https://wiki.zfin.org/display/general/ZFIN+Zebrafish+Nomenclature+Guidelines) are different from those of humans/non-human primates (http://www.genenames.org). All abbreviations were defined at their initial usage in this report.

## Materials and methods

### Fish and tissue sampling

Specimens of *A. testudineus* (20–50 g) were procured from a fish farm in Singapore. Fish specimens were acclimated and maintained as described in Chen et al. (2017). Feeding was stopped 2 days before the experiment (Tay et al., 2006). Approval to carry out the experimental procedures has been received from the Institutional Animal Care and Use Committee, National University of Singapore (IACUC 021/10 and 098/10).

Control fish (*N* = 10; *N* = 4 for molecular work, *N* = 3 each for Western blot and immunofluorescence microscopy) were submerged in 25 volumes (v/w) of fresh water. For seawater exposure, fish were progressively acclimated to a daily increment in salinity from fresh water to seawater (salinity 30) through a 6-day period (day 1: fresh water, day 2: salinity 10, day 3: salinity 15, day 4: salinity 20, day 5: salinity 25, day 6: salinity 30), and then maintained in salinity 30 for 1, 3 or 6 days (*N* = 18; *N* = 12 for molecular work, *N* = 3 each for Western blot and immunofluorescence microscopy). Waters of different salinities were prepared by mixing seawater with fresh water in different proportions. Fish were subjected to 100 mmol l^−1^ NH_4_Cl in fresh water (*N* = 18; *N* = 12 for molecular work, *N* = 3 each for Western blot and immunofluorescence microscopy) following the procedures of Chen et al. (2017). Fish were anesthetized and killed before tissue sampling according to the methods described in Chen et al. (2017). Gills were excised immediately and snap-frozen in liquid nitrogen before storing at −80°C or processed for immunofluorescence microscopy as described below.

### Total RNA isolation and reverse transcription

Total RNA was extracted from gill samples and purified as described by Chen et al. (2017). RNA concentration was subsequently determined with a BioSpec-nano (Shimadzu, Tokyo, Japan) and the quality of the RNA samples determined electrophoretically before using for cDNA synthesis.

### PCR amplification

The partial *nhe3* sequence was obtained using gene-specific primers (forward: 5′-GTCCACGTCAACGAGGTC-3′ and reverse: 5′-ATTCCACTTGTCCCTCATGTAG-3′) designed from the conserved regions of various *nhe3* mRNA sequences from fish species obtained from National Centre Biotechnology Information (NCBI). The PCR reaction was prepared using the methods and thermocycling conditions stated in Chen et al. (2017). The PCR product of the estimated size was extracted and purified using the FavorPrep™ Gel Purification Mini Kit (Favorgen Biotech Corporation, Ping-Tung, Taiwan) and sequenced.

### RACE-PCR

RACE-PCR was carried out with the Advantage® 2 PCR kit (Clontech Laboratories), using gene-specific primers (5′ RACE: 5′-AGCCCGTGTCGAGGATGATTTGAG-3′ and 3′ RACE: 5′-GAACACAGGCTTCATCCTCCTCACAC-3′), which were designed based on the partial sequence of *nhe3*. The thermocycling conditions used were identical to those of Chen et al. (2017). RACE-PCR products were separated electrophoretically and multiple rounds of sequencing were performed in both directions. Analysis of the assembled sequence was performed using Bioedit v7.1.3 and the complete cDNA coding sequence of *nhe3* has been deposited to Genbank with accession number KU555941.

### Deduced amino acid sequence and phylogenetic analysis

The deduced Nhe3 amino acid sequence was aligned and compared with Nhe3/NHE3 amino acid sequences of *Oryzias latipes, Oreochromis mossambicus, Xenopus tropicalis, Homo sapiens*, and the most thoroughly characterized isoform, NHE1, from *H. sapiens*, using BioEdit. The transmembrane (TM) domains were determined using the PSIPRED secondary structure prediction server (http://bioinf.cs.ucl.ac.uk/psipred/). After aligning the sequences with ClustalX2, phylogenetic analysis was performed, using maximum likelihood analysis and 100 bootstrap replicates, with Phylip v3.6. Nhe3/NHE3 amino acid sequences of selected animal species were obtained from Genbank or UniProtKB/TrEMBL with the following accession numbers: *Oncorhynchus mykiss* Nhe3 (ABO32815.2), *Danio rerio* Nhe3a (ABU68834.1), *D. rerio* Nhe3b (ABU68830.1), *Tribolodon hakonensis* Nhe3 (BAB83083.1), *Cynoglossus semilaevis* Nhe3 (XP_008329339.1), *O. mossambicus* Nhe3 (BAF80347.1), *O. latipes* Nhe3 (XP_011479629.1), *X. tropicalis* Nhe3 (XP_004915375.1), *Hynobius nigrescens* Nhe3 (BAF76797.1), *Oryctolagus cuniculus* NHE3 (AAA31420.1), *Fukomys damarensis* NHE3 (KFO38498.1), *Rattus norvegicus* NHE3 (AAA41702.1), *Mus musculus* NHE3 (XP_006517084.1), *Macaca fascicularis* NHE3 (XP_005556582.1), *H. sapiens* NHE3 (NP_004165.2), and *Patiria pectinifera* Nhe3 (ABQ10588.1) as the outgroup.

### qPCR

Total RNA (4 μg) from gill samples was reverse transcribed, and qPCR reaction prepared and performed as described by Chen et al. (2017), using 0.2 μmol l^−1^ of gene-specific qPCR primers (forward: 5′-ACAGTAGAAACACAACAAGGCA-3′ and reverse: 5′-CACCACTCTCACCTTCATCAG-3′). Threshold cycle, as C_t_ value, was collected at each elongation step. Melt curve analysis was performed, and the PCR products were separated in a 2% agarose gel to verify the presence of a single product.

Absolute quantification was adopted in this study, as it was crucial to assess the transcript levels of *nhe3* across different experimental conditions in the gills of *A. testudineus*, which was not possible to achieve through relative quantification. In order to produce a pure amplicon (standard) of a defined region of *nhe3* cDNA from the gills of *A. testudineus*, PCR was performed with the gene-specific qPCR primers and cDNA as a template according to the method described by Gerwick et al. (2007). After gel separation and purification as described above, the *nhe3* fragment was cloned using pGEM®-T Easy vector (Promega Corporation, Madison, WI, USA) and the presence of the insert was confirmed by sequencing. The recombinant clone was quantified using the BioSpec-nano (Shimadzu), and serially diluted (from 10^6^ to 10^2^ copies/2 μl) to obtain the standard curve using the default settings of the StepOnePlus™ Software v2.3 (Thermo Fisher Scientific, Waltham, MA, USA). The PCR efficiency for *nhe3* was 90.8%. The quantity of transcript in each sample was determined from the linear regression line derived from the standard curve.

### Western blot

A rabbit polyclonal anti-Nhe3 antibody was designed against residues 736–749 (GKSPDRSRSYHSGD) of the deduced Nhe3 sequence from *A. testudineus* (GenScript, Piscataway, NJ, USA). Anti-Nhe3 (10 μg) was pre-incubated with the immunizing peptide of Nhe3 (150 μg) in a final volume of 10 ml at 25°C for 1 h to validate the specificity of the antibody.

Gill samples were prepared for Western blotting following the methods described in Chen et al. (2017). Briefly after homogenization, the homogenate was centrifuged and protein concentration of the supernatant determined (Bradford, 1976) before diluting to 5 μg μl^−1^ with Laemmli buffer (Laemmli, 1970). Proteins (50 μg) were separated by SDS-PAGE [8% acrylamide (for anti-Nhe3) or 12% acrylamide (for anti-actin) as the resolving gel and 4% acrylamide as the stacking gel] and transferred onto a PVDF membrane. The detection of Nhe3 and actin (Developmental Studies Hybridoma Bank, Iowa City, IA, USA) were performed using Pierce™ Fast Western kit, SuperSignal™ West Pico Substrate (Thermo Fisher Scientific), following the manufacturer's instruction. After transfer, primary antibody incubation (anti-Nhe3 antibody, 1:500 dilution or anti-actin antibody, 1:10,000 dilution) was performed as described by Chen et al. (2017) and subsequently incubated with an optimized concentration of anti-rabbit or anti-mouse horseradish peroxidase-conjugated secondary antibody, respectively, for 15 min at 25°C. Bands for both Nhe3 and actin were visualized by chemiluminescence and processed following the methods described by Chen et al. (2017). Band intensities were quantified using ImageJ (version 1.41, NIH) and the results (arbitrary densitometric units) were presented as the relative protein abundance of Nhe3 normalized with the protein abundance of actin.

### Immunofluorescence microscopy

Paraffin sections of gill samples from *A. testudineus* were prepared following the methods described by Chen et al. (2017). Antigen retrieval was carried out with 0.05% citraconic anhydride in a boiling water bath (Namimatsu et al., 2005). After blocking with 5% BSA/TPBS, sections were incubated with: (1) the rabbit anti-NKAαRb1 polyclonal antibody (a pan-specific antibody originally designed by Ura et al., 1996 for labeling Nka α-subunits) co-labeled with the mouse anti-Nkaα1b monoclonal antibody (Abmart, Shanghai, China; Ching et al., 2013) or the mouse anti-Nkaα1c polyclonal antibody (Genscript; Chen et al., 2017) to examine whether Nkaα1b and Nkaα1c were present in different types of ionocyte, (2) the custom-made rabbit anti-Nhe3 antibody (Genscript) co-labeled with the mouse anti-Nkaα1a monoclonal antibody (Abmart), or the mouse anti-Nkaα1b monoclonal antibody (Abmart), or the mouse anti-Nkaα1c polyclonal antibody (Genscript) to determine the type of Nka α-isoform associated with Nhe3 in the gills of *A. testudineus*, and (3) the mouse anti-Nkaα1b antibody (Abmart) or the mouse anti-Nkaα1c antibody (Genscript) co-labeled with the rabbit anti-Rhcg1 antibody (kindly provided by S. Hirose) or the rabbit anti-Rhcg2 antibody (Genscript; Chen et al., 2017) to examine if they were localized to the same type of ionocyte.

The rabbit polyclonal anti-Rhcg1 antibody was raised against residues 425–488 of *D. rerio* Rhcg1 (Nakada et al., 2007). The anti-Nkaα1a and anti-Nkaα1b antibodies have been validated to be isoform-specific with no cross-reactivity previously by Ching et al. (2013) while the specificities of the anti-Rhcg1 and anti-Rhcg2 antibodies have been validated formerly through a peptide competition test by Chen et al. (2017). To validate the specificity of anti-Nkaα1c binding in this study, anti-Nkaα1c (6.25 μg) was pre-incubated with the immunizing peptide of Nkaα1c (62.5 μg) provided by Genscript, in a total volume of 10 ml at 25°C for 1 h before performing Western blotting and the results are shown in Supplementary Figure S1.

The anti-Nkaα1a and anti-Nkaα1b antibodies were diluted 1:1,000 in 5% BSA/TPBS (blocking buffer). The anti-Rhcg1 and anti-Rhcg2 antibodies were diluted 1:500 in 5% BSA/TPBS. The anti-Nkaα1c antibody was diluted 1:200 in 5% BSA/TPBS and the anti-Nhe3 antibody was diluted 1:100 in 5% BSA/TPBS. Secondary antibody incubations were performed as described by Chen et al. (2017). Both primary and secondary antibody incubations were performed at 37°C for 1 h. After rinsing thrice with TPBS, sections were mounted and cured in ProLong® Gold Antifade mounting medium (Thermo Fisher Scientific) before viewing and images were captured following the methods described in Chen et al. (2017). Adjustments, if necessary, were done to the entire image without altering the integrity of the data.

### Statistical analyses

All analyses were performed using the statistics software, SPSS version 18 (IBM Corporation, Armonk, NY, USA). Levene's test was used to verify the homogeneity of variances. One-way analysis of variance was performed with multiple comparisons of means by Tukey's test (for equal variances) or Dunnett's T3 (for unequal variances) for means acquired from absolute quantification. Differences in relative band intensities from Western blot were analyzed with Independent Samples *T*-test. All statistical tests were two-tailed and the significance level was set at *P* < 0.05.

## Results

### Sequence analysis of *nhe3*/Nhe3 and phylogenetic analysis of Nhe3

**Figure 1:**
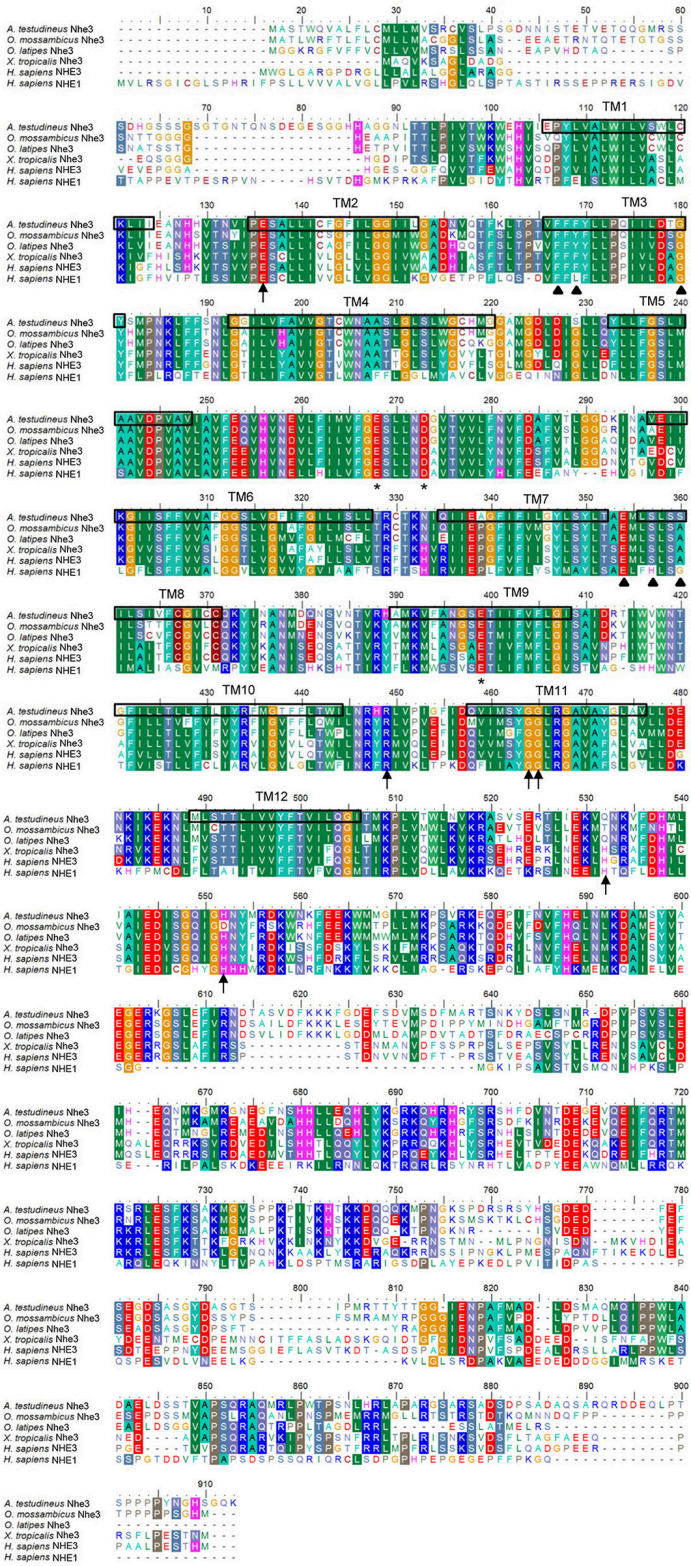
Molecular characterization of Na^+^/H^+^ exchanger 3 (Nhe3) from the gills of *Anabas testudineus*. Multiple amino acid alignment of Nhe3 from the gills of *A. testudineus*, with *Oryzias latipes* Nhe3 (XP_011479629.1), *Oreochromis mossambicus* Nhe3 (BAF80347.1), *Xenopus tropicalis* Nhe3 (XP_004915375.1), *Homo sapiens* NHE3 (NP_004165.2) and *H. sapiens* NHE1 (NP_003038.2). Asterisks represent coordinating residues for Na^+^ binding while arrows indicate residues important for coordinating H^+^ binding and intracellular pH sensing. Residues that determine the binding affinity and sensitivity to amiloride are indicated by triangles. The 12 transmembrane domains present are indicated from TM1 to TM12.

**Figure 2:**
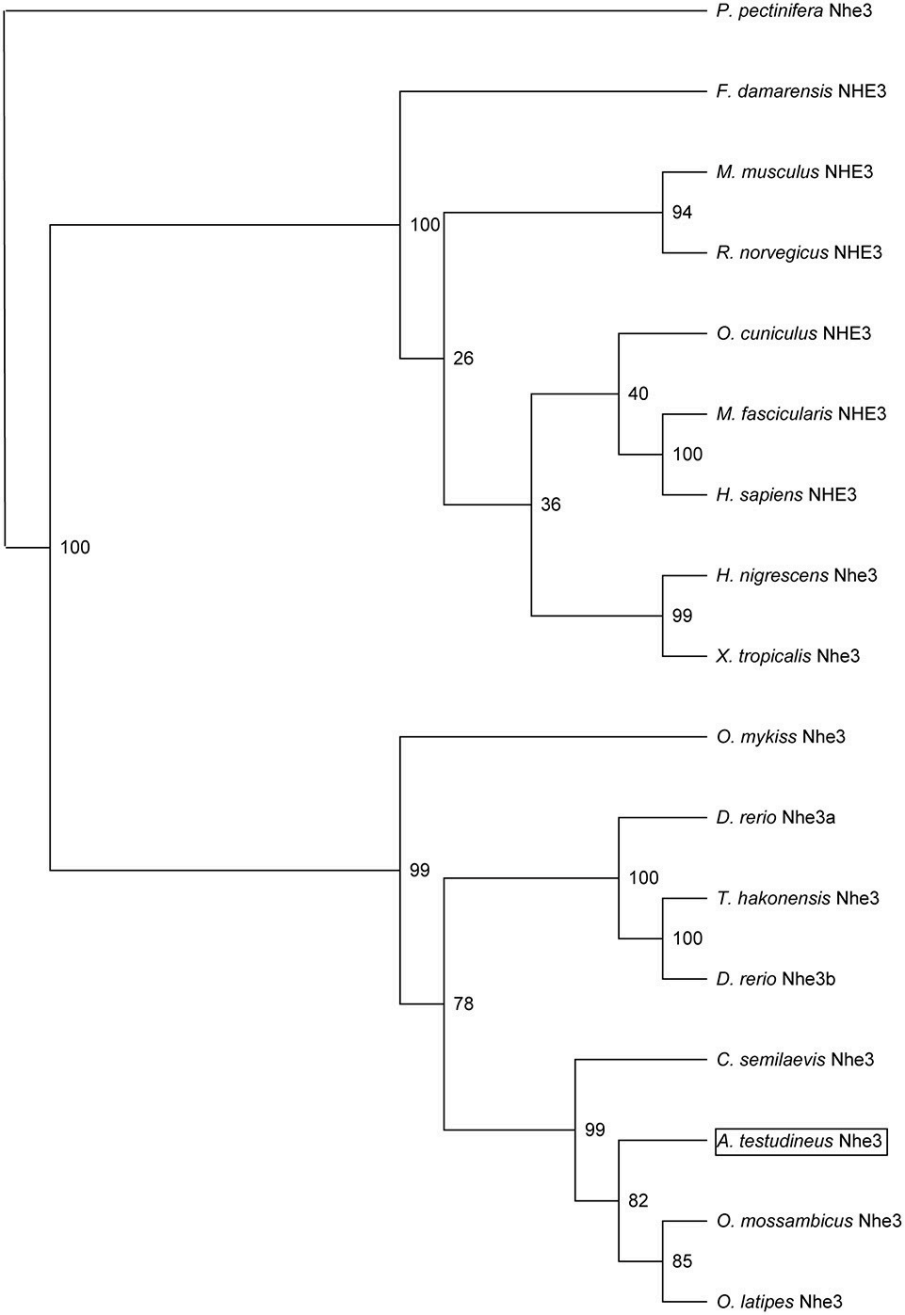
Phylogenetic analysis of Na^+^/H^+^ exchanger 3 (Nhe3) from the gills of *Anabas testudineus*. Numbers presented at each branch point represent bootstrap values from 100 replicates. Nhe3 from *Patiria pectinifera* is used as an outgroup for the phylogenetic tree.

The complete cDNA coding sequence of *nhe3* obtained from the gills of *A. testudineus* comprised 2,628 bp (Genbank accession number KU555941), coding for 876 amino acids with an estimated molecular mass of 97 kDa (Figure 1). The deduced Nhe3 sequence had 12 TM regions (Figure 1). A phylogenetic analysis revealed that Nhe3 of *A. testudineus* was more closely related to the Nhe3 sequences of teleosts than those of amphibians and mammals (Figure 2). An alignment of Nhe3 from *A. testudineus* with various animal species indicated highly conserved residues (E252, D257, and E383; corresponding to E262, D267, and E391 of human NHE1) involved in coordinating Na^+^ binding (Figure 1). Coordination of H^+^ binding and regulation of intracellular pH are known to be performed by the following residues in NHE: E131, R440, G455, G456, H480, and H500, and five of these residues (except for H480) were conserved in Nhe3 of *A. testudineus*. Nhe is a target for inhibition by amiloride (Benos, 1982); of the six residues identified as crucial for determining amiloride sensitivity in NHE1, three residues were conserved in Nhe3 of *A. testudineus* (F151, G164, and E338; corresponding to F161, G174, and E346 of human NHE1) (Figure 1). Notably, there were three amino acid substitutions (L153F, H341S, and G344S; corresponding to L163, H349, and G352 of human NHE1) within the amiloride-binding site in the Nhe3 sequence of *A. testudineus*.

### mRNA expression level of *nhe3*

**Figure 3:**
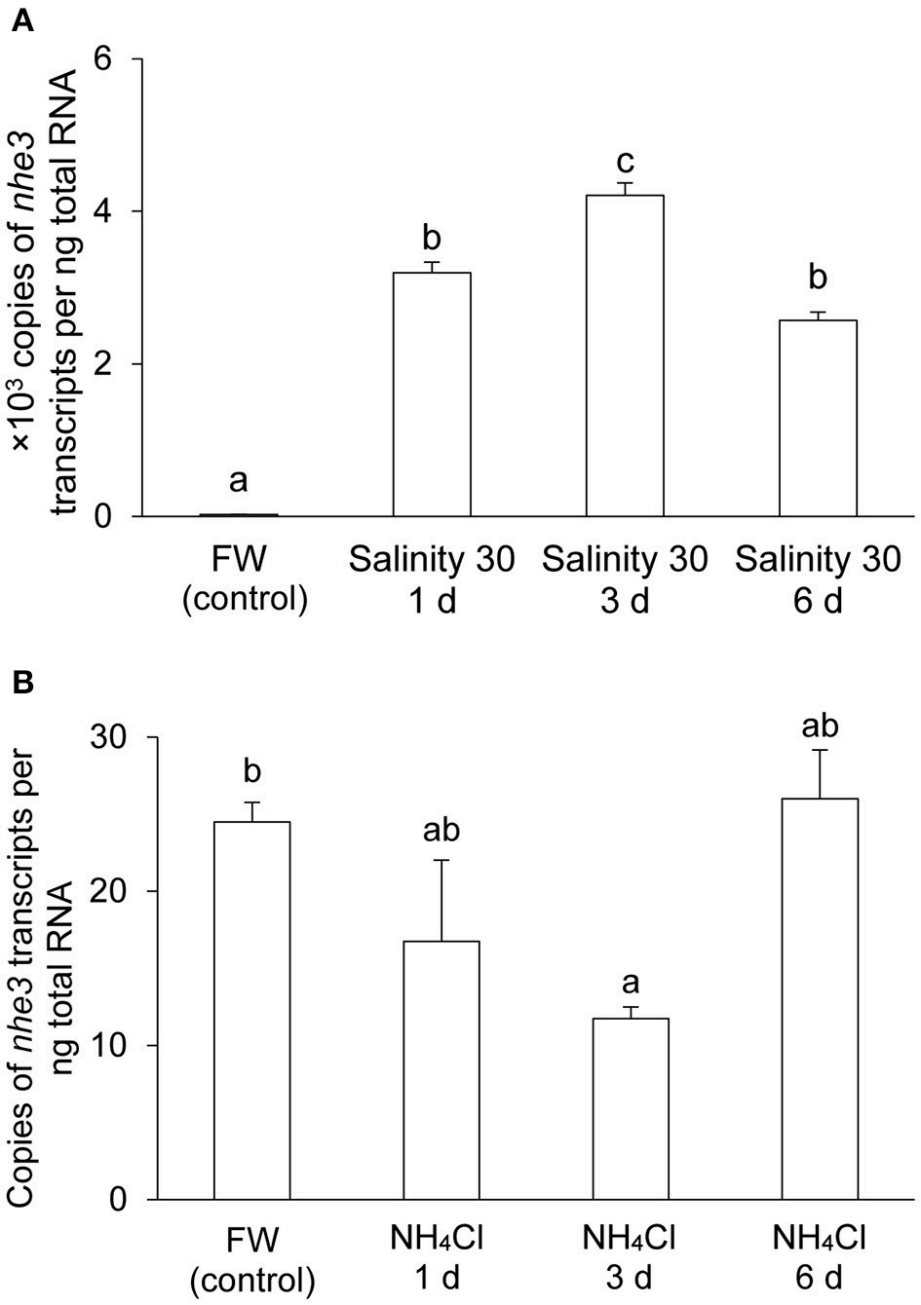
mRNA expression levels of *Na*^+^*/H*^+^ *exchanger 3* (*nhe3*) in the gills of *Anabas testudineus*. Absolute quantification of *nhe3* mRNA levels in the gills of *A. testudineus* kept in **(A)** fresh water (FW; control) or after 1, 3 or 6 days (d) of exposure to seawater (salinity 30) following a 5-day progressive increase in ambient salinity, or in **(B)** FW (control) or after 1, 3, or 6 d of exposure to 100 mmol l^−1^ NH_4_Cl. Results were presented as means + S.E.M. (*N* = 4). Different letters indicate a significant difference (*P* < 0.05).

There were significant increases in the mRNA expression levels of *nhe3* in the gills of *A. testudineus* after 1 day (by 130-fold), 3 days (by 172-fold), or 6 days (by 105-fold) of exposure to seawater, as compared to the freshwater control (Figure 3A). By contrast, a significant decrease (by 52%) in the transcript level of *nhe3* was observed in the gills of *A. testudineus* exposed to 100 mmol l^−1^ NH_4_Cl for 3 days, although it returned to control levels on the sixth day (Figure 3B).

### Protein abundance of Nhe3

**Figure 4:**
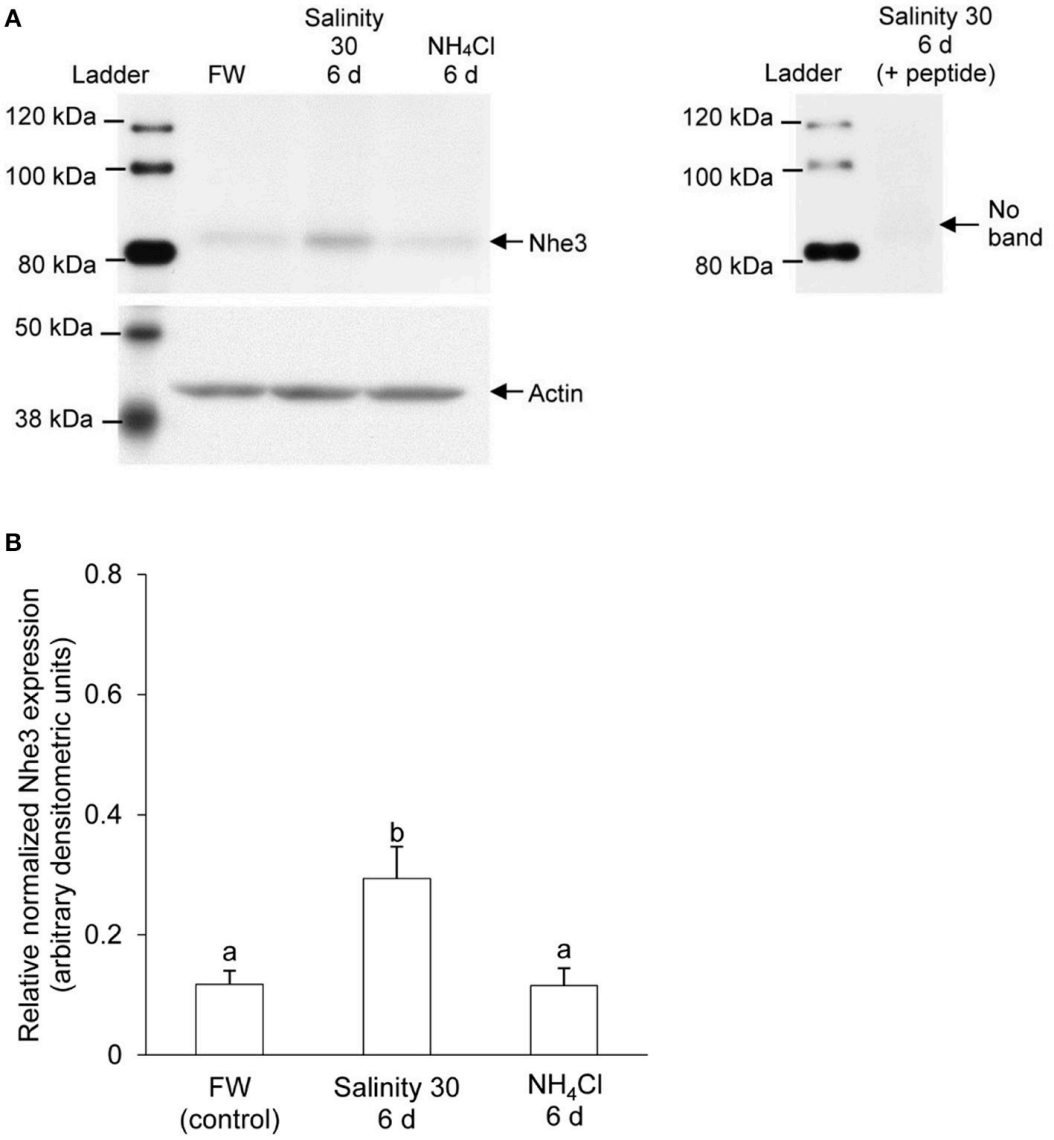
Western blotting results of Na^+^/H^+^ exchanger 3 (Nhe3) in the gills of *Anabas testudineus*. Protein abundance of Nhe3 in the gills of *A. testudineus* kept in fresh water (FW; control), after 6 days (d) of exposure to seawater (salinity 30) following a 5-day progressive increase in ambient salinity, or after 6 d of exposure to 100 mmol l^−1^ NH_4_Cl. **(A)** A representative blot of Nhe3 with actin (left), and results of the peptide competition test (right). **(B)** The protein abundance of Nhe3 normalized with respect to the protein abundance of actin. Results were presented as mean + S.E.M. (*N* = 3). Different letters indicate a significant difference (*P* < 0.05).

Immunoreactive band of Nhe3 was detected near the molecular mass of ~85 kDa which was slightly less than the expected molecular mass of 97 kDa. The protein abundance of Nhe3 increased significantly (by 2.6-fold) in the gills of *A. testudineus* exposed to seawater for 6 days (Figure 4A), but remained unchanged in the gills of fish exposed to 100 mmol l^−1^ NH_4_Cl for 6 days (Figure 4B). The validity of antibody binding was confirmed by a peptide competition test (Figure 4A).

### Immunofluorescent localization of Nhe3, Nkaα1b, Nkaα1c, Rhcg1, and Rhcg2

**Figure 5:**
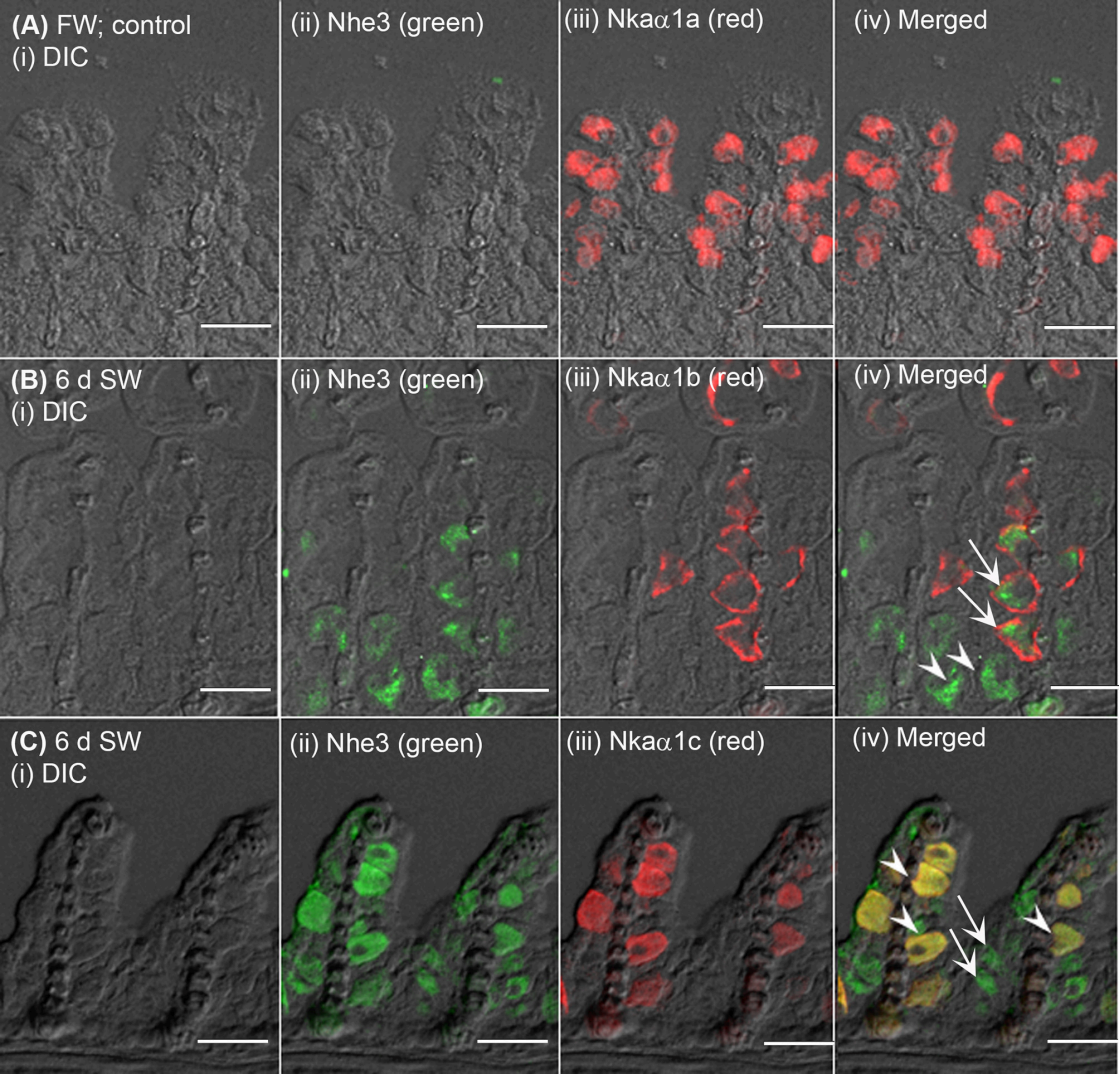
Immunofluorescent localization of Na^+^/H^+^ exchanger 3 (Nhe3) and Na^+^/K^+^-ATPase (Nka) α-subunit isoforms (Nkaα) in the gills of *Anabas testudineus* exposed to fresh water or seawater. An example of a micrograph of the gills of *A. testudineus* **(A)** stained with anti-Nhe3 and anti-Nkaα1a antibodies in the freshwater (FW; control) fish, or **(B)** stained with anti-Nhe3 and anti-Nkaα1b antibodies in fish exposed to seawater (salinity 30) for 6 days (d), or **(C)** stained with anti-Nhe3 and anti-Nkaα1c antibodies in fish exposed to seawater for 6 d. Differential interference contrast (DIC) of the filament section is shown in (i), and the immunofluorescence of an individual antibody superimposed with DIC is shown in (ii) and (iii). The merged image is presented in (iv). Arrows denote apical localization of Nhe3 while arrowheads denote basolateral localization of Nhe3. In **(B)**, arrows denote the possibly apical localization of Nhe3 in a type of ionocytes which express basolateral Nkaα1b, and arrowheads indicate the presumably basolateral Nhe3 in a type of cells which do not express Nkaα1b (these could be Nkaα1c-immunoreactive ionocytes). In **(C)**, arrows denote the expression of Nhe3 in a type of cells which do not express Nkaα1c (these could be Nkaα1b-immunoreactive ionocytes), and arrowheads indicate the co-localization of basolateral Nhe3 and basolateral Nkaα1c in a type of Nkaα1c-immunoreactive ionocytes. Scale bar: 20 μm.

**Figure 6:**
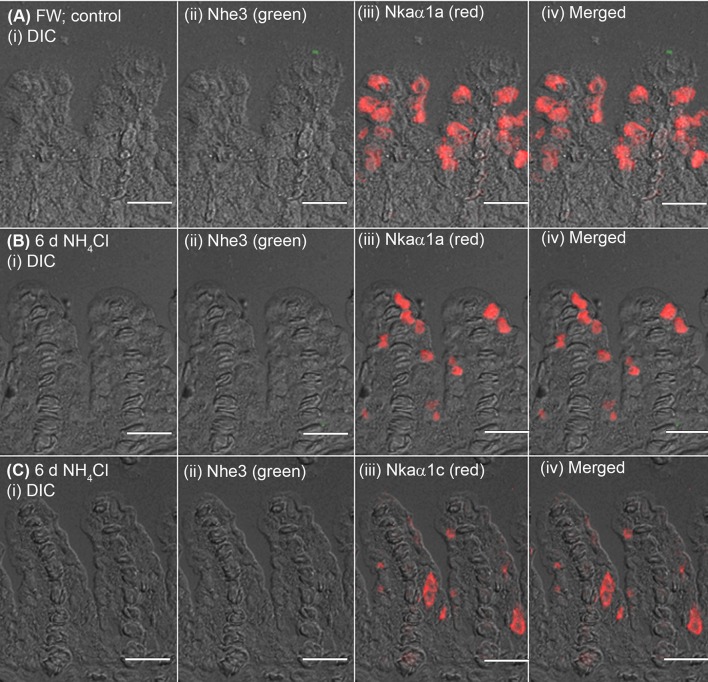
Immunofluorescent localization of Na^+^/H^+^ exchanger 3 (Nhe3) and Na^+^/K^+^-ATPase (Nka) α-subunit isoforms (Nkaα) in the gills of *Anabas testudineus* exposed to fresh water or ammonia in fresh water. An example of a micrograph of the gills of *A. testudineus* **(A)** stained with anti-Nhe3 and anti-Nkaα1a antibodies in the freshwater (FW; control) fish, or **(B)** stained with anti-Nhe3 and anti-Nkaα1a antibodies in fish exposed to 100 mmol l^−1^ NH_4_Cl in fresh water for 6 days (d), or **(C)** stained with anti-Nhe3 and anti-Nkaα1c antibodies in fish exposed to 100 mmol l^−1^ NH_4_Cl in fresh water for 6 d. Differential interference contrast (DIC) of the filament section is shown in (i), and the immunofluorescence of an individual antibody superimposed with DIC is shown in (ii) and (iii). The merged image is presented in (iv). Scale bar: 20 μm.

**Figure 7:**
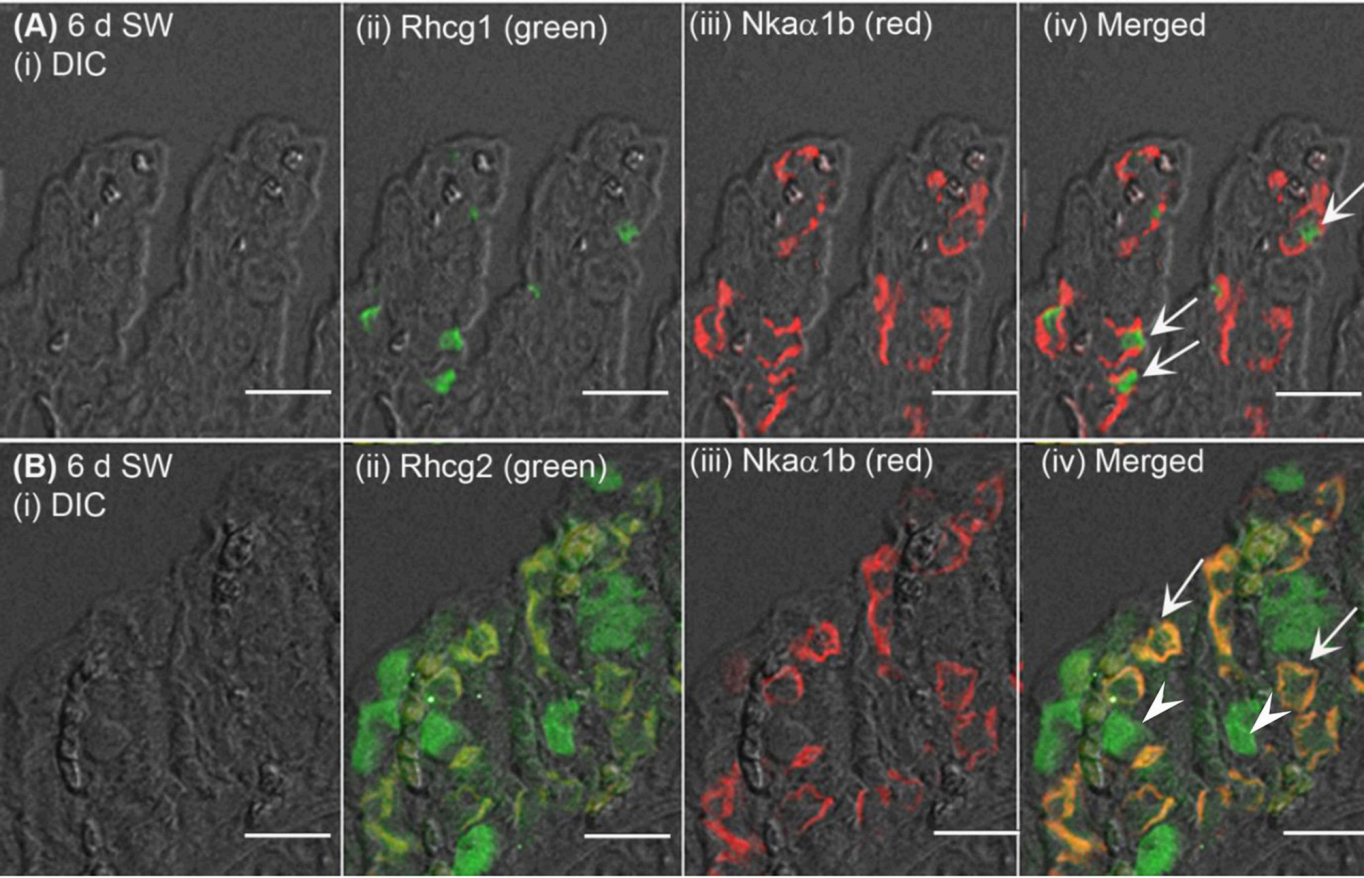
Immunofluorescent localization of Rhesus family C glycoprotein 1 (Rhcg1) or Rhcg2 with Na^+^/K^+^-ATPase (Nka) α-subunit b (Nkaα1b) in the gills of *Anabas testudineus* exposed to seawater. An example of a micrograph of the gills of *A. testudineus* **(A)** stained with anti-Rhcg1 and anti-Nkaα1b antibodies, or **(B)** stained with anti-Rhcg2 and anti-Nkaα1b antibodies in fish exposed to seawater (salinity 30) for 6 days (d). Differential interference contrast (DIC) of the filament section is shown in (i), and the immunofluorescence of an individual antibody superimposed with DIC is shown in (ii) and (iii). The merged image is presented in (iv). In **(A)**, arrows denote apical Rhcg1 in the Nkaα1b-immunoreactive ionocytes which express basolateral Nkaα1b. In **(B)**, arrows denote co-localization of basolateral Rhcg2 and basolateral Nkaα1b in the Nkaα1b-immunoreactive ionocytes, and arrowheads indicate the expression of basolateral Rhcg2 in another type of cells which do not express Nkaα1b (these could be Nkaα1c-immunoreactive ionocytes; see Figure 8). Scale bar: 20 μm.

**Figure 8:**
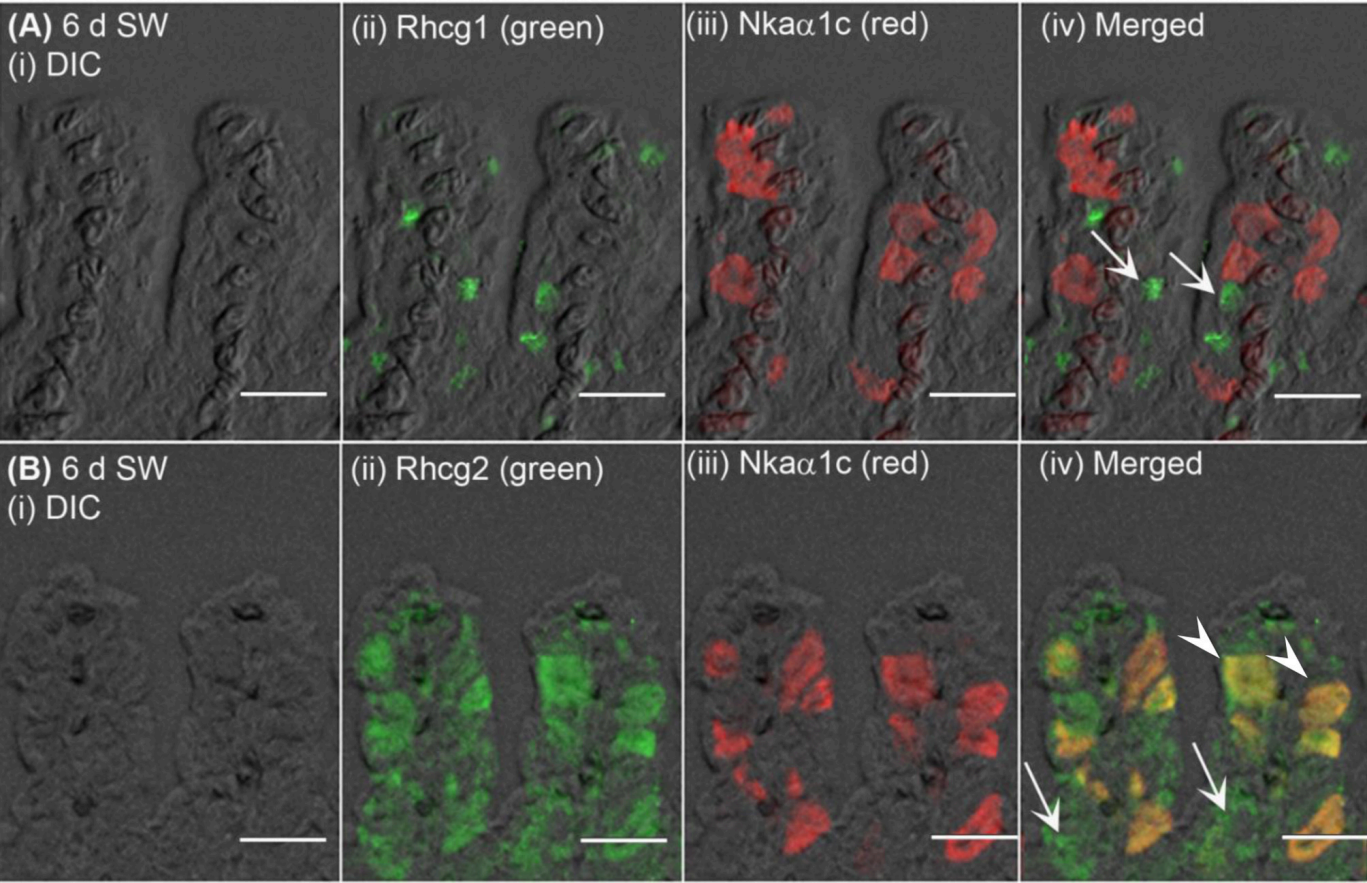
Immunofluorescent localization of Rhesus family C glycoprotein 1 (Rhcg1) or Rhcg2 with Na^+^/K^+^-ATPase (Nka) α-subunit c (Nkaα1c) in the gills of *Anabas testudineus* exposed to seawater. An example of a micrograph of the gills of *A. testudineus* **(A)** stained with anti-Rhcg1 and anti-Nkaα1c antibodies, or **(B)** stained with anti-Rhcg2 and anti-Nkaα1c antibodies in fish exposed to seawater (salinity 30) for 6 days (d). Differential interference contrast (DIC) of the filament section is shown in (i), and the immunofluorescence of an individual antibody superimposed with DIC is shown in (ii) and (iii). The merged image is presented in (iv). In **(A)**, arrows denote apical Rhcg1 in a type of cells (these could be Nkaα1b-immunoreactive ionocytes; see Figure 7) which does not express Nkaα1c. In **(B)**, arrowheads denote co-localization of basolateral Rhcg2 and basolateral Nkaα1c in the Nkaα1c-immunoreactive ionocytes, and arrows indicate the expression of basolateral Rhcg2 in another type of cells which do not express Nkaα1c (these could be Nkaα1b-immunoreactive ionocytes; see Figure 7). Scale bar: 20 μm.

The expression of Nhe3 was undetectable in the gills of *A. testudineus* kept in fresh water or exposed to 100 mmol l^−1^ NH_4_Cl in fresh water (Figures 5A, 6A–C). When acclimated to seawater, the gills of *A. testudineus* expressed Nkaα1b and Nkaα1c in the basolateral membranes of two distinct types of seawater-inducible ionocyte (Supplementary Figure S2). Results obtained from the gills of the seawater-exposed fish revealed for the first time that Nhe3 had a different subcellular localization in two distinct types of ionocyte which were immunoreactive to either the anti-Nkaα1b antibody (Figure 5B) or the anti-Nkaα1c antibody (Figure 5C). Nhe3 was detected in the apical membranes of a large proportion of Nkaα1b-labeled ionocytes (Figure 5B), but it was expressed in the basolateral membranes of Nkaα1c-labeled ionocytes (Figure 5C). In either case, there were ionocytes that were labeled with only Nhe3, and these ionocytes probably expressed the other seawater-specific Nkaα isoform (either Nkaα1b or Nkaα1c) in the basolateral membranes (Figures 5B,C). Additionally, the majority of the Nkaα1b-labeled ionocytes in the gills of the seawater-acclimated fish expressed Rhcg1 (Figure 7A) and Rhcg2 (Figure 7B) on the apical and basolateral membranes, respectively. By contrast, the Nkaα1c-labeled ionocytes in the gills of the seawater-acclimated fish expressed only basolateral Rhcg2 (Figure 8B) without apical Rhcg1 (Figure 8A).

## Discussion

### Molecular characterization of Nhe3 from *A. testudineus*

Similar to members of the NHE family, Nhe3 from *A. testudineus* comprised an N-terminal domain, with 12 TM regions, which is mainly responsible for the catalysis of amiloride-sensitive electroneutral Na^+^/H^+^ exchange. It also had a C-terminal domain that serves a regulatory function (Wakabayashi et al., 1992). Residues involved in the coordination of cation binding and intracellular pH regulation, which are characteristic of the well-studied human NHE1 (Murtazina et al., 2001; Wakabayashi et al., 2003; Slepkov et al., 2007), were highly conserved in the Nhe3 of *A. testudineus*.

Site-specific mutagenesis demonstrates that the acidic side chains of E262, D267, and E391 are essential for NHE activity (Murtazina et al., 2001). Mutation of E262 and D267 effectively eliminates transport activity while mutation at E391 reduces NHE activity partially, thereby showing the importance of the oxygen atom in the side chains of these negatively-charged amino acids in Na^+^ binding (Boyer, 1988; Murtazina et al., 2001). An arginine residue (A440) and two glycine residues (G455 and G456) have been identified to be critical for intracellular pH sensing as they reside in the putative “pH_i_ sensor” site in NHE1, and regulate the pH_i_ sensitivity of NHE1 in opposite directions (Wakabayashi et al., 2003). E131 is another residue crucial in regulating Na^+^ transport and the pH sensitivity of NHE1. Charge reversal mutations of E131 result in a shift in the intracellular pH dependence of NHE1 activity and eliminate NHE1 response to a hyperosmotic medium (Hisamitsu et al., 2007). As the imidazole side chains of histidine residues can be protonated, mutations at H480 and H500 in NHE3 alters the binding of protons at these positions (Cha et al., 2002). This significantly affects the pH sensitivity of the mutant NHE3 as its activity threshold is shifted to a more acidic region. Of the nine residues stated above as important for NHE activity, eight of them (with the exception of H480) were present in Nhe3 of *A. testudineus*. The replacement of H480 in Nhe3 of *A. testudineus* is probably a common phenomenon as substitution at this position was observed in all the Nhe3 sequences of teleosts examined here. These highly conserved residues further confirm the importance of the Na^+^ and H^+^ binding sites and the “pH_i_ sensor” site in Nhe3 of *A. testudineus* and suggests that the ion exchange activity of Nhe3 in *A. testudineus* probably functions via a similar mechanism of action.

Different NHE isoforms display varying affinity for its inhibitors, amiloride and its analogs, in the following order of sensitivity: NHE1 ≥ NHE2 > NHE5 > NHE3 > NHE4 (Masereel et al., 2003; Orlowski and Grinstein, 2004). Six amino acids (F161, L163, G174, E346, G352, and H349) are implicated as residues important for inhibitor binding and hence, NHE1 function. L167F (corresponding to L163 in human NHE1) mutation in hamster increases the resistance of NHE1 to amiloride but has no effect on Na^+^ transport (Counillon et al., 1993). In fact, the leucine at this position of all the Nhe3/NHE3 sequences examined in this study was replaced with either phenylalanine or tyrosine. Hence, this difference could be characteristic of Nhe3/NHE3 and contribute to the difference in amiloride affinity between NHE1 and NHE3 (Masereel et al., 2003; Orlowski and Grinstein, 2004). Likewise, the substitution of H349 with serine was common among all the Nhe3/NHE3 examined in this study, and H349S in NHE1 is known to cause a change in its amiloride sensitivity (Wang et al., 1995).

### The branchial *nhe3*/Nhe3 expression levels are low in freshwater *A. testudineus*, but increase drastically upon seawater acclimation

Two working models have been proposed for apical Na^+^ uptake in the gills of freshwater teleosts, depending on the fish species and the environment of its habitat: (1) an apical H^+^-ATPase electrically coupled to Na^+^ uptake via ENaC and (2) an electroneutral exchange of Na^+^ and H^+^ by apical Nhe (Hwang and Lee, 2007). For instance, stenohaline zebrafish depends largely on H^+^-ATPase for Na^+^ uptake, but the euryhaline medaka relies on Nhe3 instead (Guh et al., 2015). A relatively high expression of Nhe3 in relation to Na^+^ uptake in a freshwater environment have been confirmed in many teleosts (Wu et al., 2010; Kumai and Perry, 2011; Shih et al., 2012). By contrast, the expression of *nhe3*/Nhe3 was weak in the gills of freshwater *A. testudineus*. Hence, it is uncertain whether Nhe3 plays a significant role in branchial Na^+^ absorption in *A. testudineus* in a hypoosmotic environment. This could perhaps be elucidated in the future by examining the effects of fresh water with low [Na^+^] on the expression levels of *nhe3*/Nhe3 in its gills.

By contrast, the expression levels of branchial *nhe3*/Nhe3 increased substantially in the seawater-acclimated *A. testudineus*. Although the observed molecular mass (85 kDa) for Nhe3 in *A. testudineus* is slightly smaller than the estimated size (97 kDa), it is not an uncommon phenomenon. “Gel-shifting” is usually observed for membrane proteins and can be attributed to the effects of differential detergent binding and folding when the protein is electrophoresed by SDS-PAGE (Rath et al., 2009). The increase in the expression of *nhe3*/Nhe3 in the gills of *A. testudineus* during seawater exposure is unsurprising as branchial Nhe3 immunoreactivity has been detected in many fish species acclimated to seawater (Evans et al., 2005; Watanabe et al., 2008; Hwang et al., 2011; Christensen et al., 2012; Seo et al., 2013), thereby implying its involvement in H^+^ secretion during acid-base regulation in a hyperosmotic environment (Claiborne et al., 2002; Choe et al., 2005). Increases in respiratory and metabolic rates in a hyperosmotic environment can result in acidosis and hence, the prompt elimination of any excess H^+^ from the system via Nhe3 is essential for the maintenance of acid-base balance (Seo et al., 2013). The uptake of Na^+^ in exchange for the excretion of H^+^ would undoubtedly aggravate the ionic load that a fish in seawater will encounter. However, the resulting influx of Na^+^ may account for only a small portion of the total intracellular concentration of Na^+^ and thus, may be “worth” the additional energetic costs to maintain acid-base balance (Evans, 1984). Furthermore, the high Na^+^ concentrations in the ambient seawater would favor Na^+^/H^+^ exchange (Potts, 1994), down a favorable Na^+^ concentration gradient generated by the basolateral Nka (Choe et al., 2002).

### Nkaα1b-immunoreactive ionocytes and Nkaα1c-immunoreactive ionocytes may have different physiological functions in seawater

The gills of the seawater-acclimated *A. testudineus* express two distinct types of seawater-inducible Nkaα-immunoreactive ionocyte labeled with either Nkaα1b or Nkaα1c (Supplementary Figure S2), which probably serve disparate physiological functions in seawater. While the Nkaα1c-immunoreactive ionocytes also express basolateral Nkcc1a and apical Cftr indicating that they are responsible for active salt excretion, the Nkaα1b-immunoreactive ionocytes lack Nkcc1a and Cftr, suggesting that they are involved in some other physiological processes which have yet to be defined (unpublished findings from X.L. Chen and Y.K. Ip). Of note, it has been established that *A. testudineus* increases the production and excretion of ammonia when exposed to seawater (Chang et al., 2007). The energy required for increased syntheses of certain branchial proteins and transport of ions during seawater acclimation can be supported by increases in protein degradation and amino acid catabolism. It is possible that the Nkaα1b-immunoreactive ionocytes take part in ammonia excretion if they express apical Nhe3 together with some sort of ammonia transporters.

### In seawater, apical Nhe3 can facilitate ammonia excretion in Nkaα1b-immunoreactive ionocytes which co-express apical Rhcg1 and basolateral Rhcg2

Ammonia excretion in fishes is achieved largely through the transport of NH_3_ down a favorable P_NH3_ gradient across the branchial epithelium, with the excreted NH_3_ being “trapped” by H^+^ excreted through the apical H^+^-ATPase and/or Nhe (Weihrauch et al., 2009; Wright and Wood, 2009; Hwang et al., 2011). Using specific morpholino oligonucleotides to knock down Rhcg1 translation in zebrafish embryos, Shih et al. (2008) showed that ${\text{NH}}_{4}^{+}$ efflux occurred favorably in H^+^-ATPase-rich cells and there was a drop in ${\text{NH}}_{4}^{+}$ efflux upon knockdown of Rhcg1 or H^+^-ATPase, or inhibition of H^+^-ATPase with bafilomycin or Nhe with EIPA. However, in some teleosts such as the mummichog (Edwards et al., 2005) and medaka (Wu et al., 2010), H^+^-ATPase is not the key player in H^+^ secretion. In fact, it has been demonstrated in embryos of medaka that Nhe3 and Rhcg1 were co-localized to a type of ionocyte, and ammonia excretion is inhibited by EIPA, but not bafilomycin, implying that Nhe3 plays a critical role in ammonia excretion (Wu et al., 2010). Using a scanning ion-selective electrode to measure H^+^ gradients, Liu et al. (2013) detected an acidic boundary layer at the yolk-sac surface of seawater-acclimated medaka larvae. They demonstrated that *nhe3, rhbg, rhcg1*, and *rhcg2* expression levels were up-regulated in the medaka larvae exposed to ammonia in seawater, suggesting that these transporters were involved in H^+^-facilitated ammonia excretion. Furthermore, Rhcg1 and Nhe3 have been co-localized in Nkaα-immunoreactive ionocytes in the gill, kidney, and skin of the mangrove killifish, whereby the apical Nhe3 apparently facilitates ammonia excretion (Cooper et al., 2013). More importantly, there are evidence to support a positive correlation between ${\text{NH}}_{4}^{+}$ excretion and Na^+^ uptake in fish gill and skin (Shih et al., 2008; Wu et al., 2010; Cooper et al., 2013; Liu et al., 2013).

As expected, Nhe3 was localized to the apical membrane of the Nkaα1b-immunoreactive ionocytes, which co-expressed apical Rhcg1 and basolateral Rhcg2, in the gills of *A. testudineus* exposed to seawater. Therefore, it is logical to infer that H^+^-facilitated ammonia excretion occurs in seawater-exposed *A. testudineus* to augment increased ammonia production, which does not occur in the freshwater control. In seawater, NH_3_ in the blood probably enters the ionocyte through the basolateral Rhcg2 and exits via the apical Rhcg1 to the external medium. The excreted NH_3_ can be “trapped” as ${\text{NH}}_{4}^{+}$ by the H^+^ excreted through the apical Nhe3. This generates an outwardly-directed ΔP_NH3_ to facilitate branchial ammonia excretion in *A. testudineus* during seawater exposure. Nkaα1b is essential to transport the intracellular Na^+^ to the blood, in order to maintain intracellular Na^+^ homeostasis and provide the driving force necessary for Nhe3 to function. The excess Na^+^ would finally exit the branchial epithelium paracellularly. In this way, *A. testudineus* is able to effectively excrete ammonia and regulate the intracellular pH of those ionocytes participating in the process.

### Nhe3 is uniquely localized to the basolateral membrane of the Nkaα1c-immunoreactive ionocytes in *A. testudineus* exposed to seawater

In fish gills, Nhe3 is usually localized to the apical membranes of ionocytes, and basolateral Nhe3 localization has not been reported before. This is the first report on Nhe3 being expressed in different membranes (apical or basolateral) of two different types of ionocyte in the gills of a fish. Of note, NHE1 is ubiquitously expressed in the basolateral membrane of epithelial cells (Donowitz et al., 2013) including branchial ionocytes of fishes (Claiborne et al., 1999). However, the Nhe isoform localized to the basolateral membrane of the Nkaα1c-immunoreactive ionocytes in *A. testudineus* could not be Nhe1 as the epitope for Nhe3 was uniquely designed against the deduced amino acid sequence of Nhe3, and did not match the Nhe1 sequence obtained from the gills of *A. testudineus* (X.L. Chen and Y.K. Ip, unpublished results). Furthermore, based on the NCBI non-redundant protein database, the epitope sequence selected to raise the custom-made anti-Nhe3 antibody displayed high similarity with equivalent sequences of Nhe3/NHE3 from various animals, as revealed by the top 10 hits of the BLAST results (Table 1). Although the Nkaα1c-labeled ionocytes displayed basolateral Rhcg2-labeling, the lack of apical Rhcg1-labeling indicates that they do not have the same physiological function as the Nkaα1b-immunoreactive ionocytes. At present, the exact function of basolateral Nhe3 in the Nkaα1c-immunoreactive ionocytes of the seawater-exposed *A. testudineus* is unknown, but we speculate that it could serve to regulate blood pH and maintain pH homeostasis in the Nkaα1c-immunoreactive ionocytes of *A. testudineus*.

**Table 1:** The top 10 results from a protein BLAST (BLASTP; with “automatically adjust parameters for short input sequences”) of the epitope sequence (residues 736-749 of the Nhe3 sequence from *Anabas testudineus*; GKSPDRSRSYHSGD) selected to raise the anti-Nhe3 antibody.

**No**.	**Description**	**Accession number**	**Total score**	***E*-value**	**Query coverage (%)**	**Identity (%)**
1	Sodium/hydrogen exchanger 3 [*Larimichthys crocea*]	KKF31822.1	36.7	0.15	100	86
2	PREDICTED: sodium/hydrogen exchanger 3 [*Larimichthys crocea*]	XP_010740588.2	36.7	0.15	100	86
3	NHE3 [*Sciaenops ocellatus*]	AIL54063.1	36.7	0.15	100	86
4	PREDICTED: sodium/hydrogen exchanger 3-like isoform X1 [*Hippocampus comes*]	XP_019721774.1	32.0	6.9	92	77
5	PREDICTED: sodium/hydrogen exchanger 3-like isoform X2 [*Hippocampus comes*]	XP_019721775.1	32.0	6.9	92	77
6	DNA polymerase lambda [*Hypsibius dujardini*]	OQV26010.1	32.0	6.9	100	79
7	PREDICTED: sodium/hydrogen exchanger 3 isoform X1 [*Lates calcarifer*]	XP_018555265.1	31.6	9.8	100	79
8	PREDICTED: sodium/hydrogen exchanger 3 isoform X2 [*Lates calcarifer*]	XP_018555270.1	31.6	9.8	100	79
9	PREDICTED: sodium/hydrogen exchanger 3 isoform X3 [*Lates calcarifer*]	XP_018555273.1	31.6	9.8	100	79
10	Hypothetical protein SAMN05216571_103203 [*Halomonas taeanensis*]	SDF96243.1	31.2	14	100	67

### Nhe3 does not take part in active ${\text{NH}}_{4}^{+}$ excretion in the gills of *A. testudineus* exposed to ammonia in fresh water

During emersion or ammonia exposure, active ammonia excretion is one of the most effective ways to ameliorate ammonia toxicity in fishes, as it can maintain low internal ammonia concentrations and prevent the brain from ammonia intoxication (see Chew et al., 2006; Ip and Chew, 2010; Chew and Ip, 2014 for reviews). Some air-breathing fishes can actively excrete ammonia through their gills; these include the climbing perch (Tay et al., 2006), the giant mudskipper (*Periophthalmodon schlossei*; Randall et al., 1999; Chew et al., 2003, 2007; Ip et al., 2004a) and the African sharptooth catfish (Ip et al., 2004b). *Periophthalmodon schlossei* is capable of lowering the pH of the external medium (Chew et al., 2003, 2007), and branchial H^+^ excretion can prevent a back flux of NH_3_ during active ${\text{NH}}_{4}^{+}$ excretion (Ip et al., 2004a). However, there are differences in the mechanisms of active ammonia excretion between the gills of *A. testudineus* (Loong et al., 2011; Ip et al., 2012a,b) and the gills of *P. schlosseri* (Chew et al., 2014b,a; Chew and Ip, 2017), as the former is incapable of lowering the pH of the external medium in response to environmental ammonia (Ip et al., 2012b). The current model of active branchial ammonia excretion in *A. testudineus* denotes that ammonia may permeate through the basolateral Nkcc1 (Loong et al., 2011) or the basolateral Rhcg2 (Chen et al., 2017) and exit the Nkaα1c-immunoreactive ionocytes as ${\text{NH}}_{4}^{+}$ through the apical Rhag (Chen et al., 2017), driven by a trans-apical membrane electrical potential generated by the efflux of ${\text{HCO}}_{3}^{-}$ or Cl^−^ via the apical Cftr (Ip et al., 2012b).

Indeed, our results indicate that Nhe3 is unlikely to play a significant role in active ${\text{NH}}_{4}^{+}$ transport in the gills of *A. testudineus*. The mRNA expression levels of *nhe3* in the gills of *A. testudineus* remained low during 6 days of exposure to 100 mmol l^−1^ NH_4_Cl in fresh water, with a significant decrease on day 3. The low branchial expression of *nhe3* (<30 copies per ng total RNA) implies that Nhe3 is unlikely to be of physiological significance. This is further supported by the lack of significant changes on the protein abundance of branchial Nhe3, and the undetectable level of apical Nhe3 in the branchial epithelium, in *A. testudineus* exposed to ammonia. Taken together, our results corroborate the report on the inability of *A. testudineus* to lower the pH of the ambient water during ammonia exposure (Ip et al., 2012b).

## Summary

Our results indicate that Nhe3 does not serve a physiological function in the gills of freshwater *A. testudineus*, but it can be involved in increased ammonia excretion in fish during exposure to seawater, as its expression is inducible by seawater and is localized to the apical membrane of the Nkaα1b-immunoreactive ionocytes. By contrast, Nhe3 is localized to the basolateral membrane of the Nkaα1c-immunoreactive ionocytes which are involved in active salt excretion during seawater exposure. Efforts should be made in the future to elucidate the function of basolateral Nhe3 in the Nkaα1c-immunoreactive ionocytes of *A. testudineus*.

## Author contributions

YI designed the experiments. XC and BZ performed the experiments and analyzed the data. XC and YI wrote the manuscript. XC, YC, JO, WW, and SC participated in animal subjection and sample collection. SL and YI were involved in the analysis of data and approval of the manuscript.

### Conflict of interest statement

The authors declare that the research was conducted in the absence of any commercial or financial relationships that could be construed as a potential conflict of interest.
