# Supplementary material for: Na+/H+ Exchanger 3 Is Expressed in Two Distinct Types of Ionocyte, and Probably Augments Ammonia Excretion in One of Them, in the Gills of the Climbing Perch Exposed to Seawater
Source: Front Physiol. 2017 Nov 2;8:880. doi: 10.3389/fphys.2017.00880 (PMC5701670; doi:10.3389/fphys.2017.00880)

Supplementary Figure S1. (A) A representative blot of Na^+/K^+-ATPase α1c (Nkaα1c) using the custom-made anti-Nkaα1c antibody. **(B)** A representative blot of Nkaα1c using the anti-Nkaα1c antibody pre-incubated with the immunizing peptide in the peptide competition test.

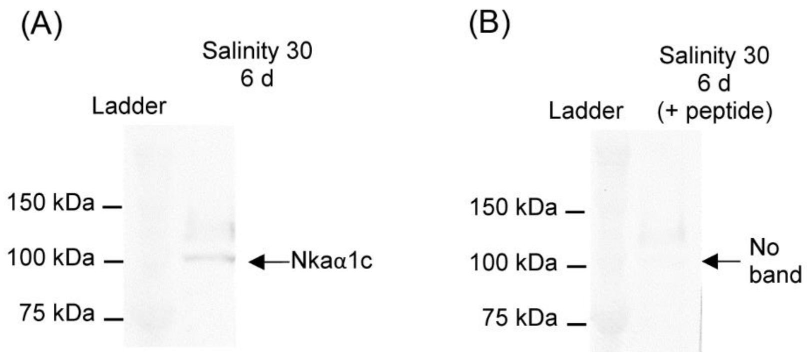

Supplement: Supplementary file 1 [file Image1.PDF]
