# Supplementary material for: Na+/H+ Exchanger 3 Is Expressed in Two Distinct Types of Ionocyte, and Probably Augments Ammonia Excretion in One of Them, in the Gills of the Climbing Perch Exposed to Seawater
Source: Front Physiol. 2017 Nov 2;8:880. doi: 10.3389/fphys.2017.00880 (PMC5701670; doi:10.3389/fphys.2017.00880)

Supplementary Figure S2. Immunofluorescent labelling of Na^+/K^+-ATPase α-subunit (Nkaα) in the gills of *Anabas testudineus* exposed to seawater (salinity 30) for 6 days (d) using the commercially available anti-NKAαRb1 antibody which is known to react comprehensively with NKAα, the custom-made anti-Nkaα1b antibody which binds specifically with Nkaα1b, or the custom-made anti-Nkaα1c antibody which binds specifically with Nkaα1c. An example of micrographs of two consecutive sections of the gills of *A. testudineus* **(A)** stained with anti-NKAαRb1 and anti-Nkaα1b antibodies or **(B)** stained with anti-NKAαRb1 and anti-Nkaα1c antibodies. Arrows in **(A)** denote the ionocytes expressing Nkaα1b while the arrowheads in **(A)** indicate the ionocytes that presumably express Nkaα1c (refer to **B**). Arrowheads in **(B)** denote the ionocytes expressing Nkaα1c while the arrows in **(B)** indicate the ionocytes that express presumably Nkaα1b (refer to **A**). Scale bar: 20 µm.

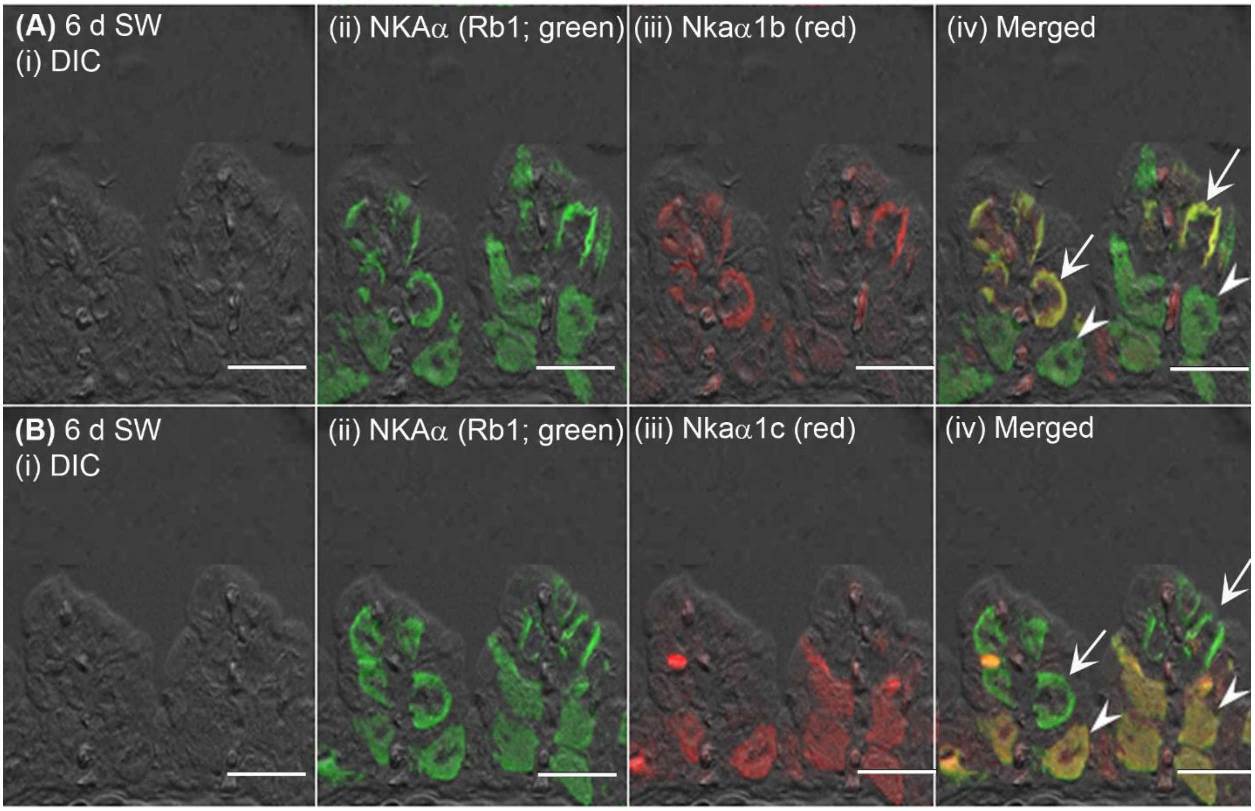

Supplement: Supplementary file 2 [file Image2.PDF]
